# Characterizing the Followers and Tweets of a Marijuana-Focused Twitter Handle

**DOI:** 10.2196/jmir.3247

**Published:** 2014-06-27

**Authors:** Patricia Cavazos-Rehg, Melissa Krauss, Richard Grucza, Laura Bierut

**Affiliations:** ^1^Washington University School of MedicineSt. Louis, MOUnited States

**Keywords:** Twitter, social media, marijuana

## Abstract

**Background:**

Twitter is a popular social media forum for sharing personal experiences, interests, and opinions. An improved understanding of the discourse on Twitter that encourages marijuana use can be helpful for tailoring and targeting online and offline prevention messages.

**Objectives:**

The intent of the study was to assess the content of “tweets” and the demographics of followers of a popular pro-marijuana Twitter handle (@stillblazingtho).

**Methods:**

We assessed the sentiment and content of tweets (sent from May 1 to December 31, 2013), as well as the demographics of consumers that follow a popular pro-marijuana Twitter handle (approximately 1,000,000 followers) using Twitter analytics from Demographics Pro. This analytics company estimates demographic characteristics based on Twitter behavior/usage, relying on multiple data signals from networks, consumption, and language and requires confidence of 95% or above to make an estimate of a single demographic characteristic.

**Results:**

A total of 2590 tweets were sent from @stillblazingtho during the 8-month period and 305 (11.78%) replies to another Twitter user were excluded for qualitative analysis. Of the remaining 2285 tweets, 1875 (82.06%) were positive about marijuana, 403 (17.64%) were neutral, and 7 (0.31%) appeared negative about marijuana. Approximately 1101 (58.72%) of the positive marijuana tweets were perceived as jokes or humorous, 340 (18.13%) implied that marijuana helps you to feel good or relax, 294 (15.68%) mentioned routine, frequent, or heavy use, 193 (10.29%) mentioned blunts, marijuana edibles, or paraphernalia (eg, bongs, vaporizers), and 186 (9.92%) mentioned other risky health behaviors (eg, tobacco, alcohol, other drugs, sex). The majority (699,103/959,143; 72.89%) of @stillblazingtho followers were 19 years old or younger. Among people ages 17 to 19 years, @stillblazingtho was in the top 10% of all Twitter handles followed. More followers of @stillblazingtho in the United States were African American (323,107/759,407; 42.55%) or Hispanic (90,732/759,407; 11.95%) than the Twitter median average (African American 22.4%, inter-quartile ratio [IQR] 5.1-62.5%; Hispanic 5.4%, IQR 3.0-10.8%) and among Hispanics, @stillblazingtho was in the top 30% of all Twitter handles followed.

**Conclusions:**

Young people are especially responsive to social media influences and often establish substance use patterns during this phase of development. Our findings underscore the need for surveillance efforts to monitor the pro-marijuana content reaching young people on Twitter.

## Introduction

Social media use is common among young persons. The majority of Internet users in the United States (72%) use social media platforms like Facebook, Twitter, LinkedIn, MySpace, YouTube, and others [1]. The rate of social media use is even higher among young adults aged 18-29 years old in the United States (89%) [2,3]. Many US social media sites have high levels of user engagement: 63% of Facebook users check the site at least daily, followed by 57% of Instagram users, and 46% of Twitter users [3]. This is especially true for youth and young adults who are the most likely age group to use Twitter. Typical users of Twitter are quite young [4]: nearly half are under the age of 34 and only 30% are over 45. While Facebook continues to dominate social media engagement, more US teens rated Twitter (26%) as the most important social media site than Facebook (23%) [5]. Focus groups revealed that teens dislike the increasing adult presence, inane details, drama, and the need to maintain their reputation on Facebook, but can better express themselves on sites like Twitter [6]. Continued growth from 1.1 billion social media users worldwide in 2013 to 2.3 billion users in 2017 is projected [7].

The term “infodemiology” was coined by Eysenbach and underscores the communication patterns on the Internet that have important implications for the study of population health and public policy [8]. Emerging evidence in the infodemiology of online substance use risk behavior content that is being viewed and posted online via social media platforms is concerning. For instance, up to 83% of US college students’ social networking sites, such as Facebook and MySpace, reference alcohol use [9]. Also, a recent study found that 39% of 15-24 year olds reported having a friend who posted online pictures of themselves smoking marijuana on Facebook or MySpace [10]. In addition, findings suggest that explicit and/or illegal online content on social media is relatively common among adolescents who are 18 years of age and under. Specifically, studies of US college students have found that underage young adults commonly post pictures of themselves drinking alcohol on Facebook [11-13]. Related studies also found references to sexual risk-taking, alcohol use, and drug use behaviors on US adolescents’ (ages 16-18 years old) public online MySpace social media profiles [9,14]. Taken together, the studies indicate a high likelihood for youth and young adults to consume and create online content about risk behaviors via social media platforms.

Like Facebook and MySpace, Twitter is a popular social media forum among youth and young adults [15]. Tweets are messages that are ≤140 characters and are sent from a user profile (“handle”) to a network of “followers” who have chosen to “follow” that particular handle. Followers receive tweets in real time via mobile phones and/or email. Twitter advertises itself as a freedom of speech social media platform and seldom removes tweets that are not illegal or spam. Therefore, it is possible for tweets that encourage deleterious health behaviors to reach youth and other vulnerable populations (eg, current substance abusers); yet, the research that addresses this topic is scant. In one study that examined exposure to alcohol beverage advertisements and marketing via Twitter, it was found that youth who were not yet of the legal drinking age could easily access alcohol marketing campaigns [16]. Similarly, underage youth were able to view and post tweets that promoted trendy tobacco products like hookah and e-cigarettes [17]. In a related study, Twitter users whose tweets identified them as prescription drug abusers tended to be “socially surrounded” (via tweets) with other Twitter users who similarly Tweeted about prescription drug abuse [18]. These findings suggest that Twitter users, even those who are young in age and cannot legally purchase substances like alcohol or tobacco, engage in Twitter activities that promote substance use behaviors.

Young people are responsive to social media influences and often establish substance use patterns during this phase of development [19-21]. In fact, the Media Practice Model (MPM) was developed to explain how individuals can use social media messages for guidance on life choices and accordingly disclose information on social media that reflects actual behaviors and traits or behavioral intent [22-25]. The MPM further postulates that youth and young adults consume and engage with media based on who they are and who they want to be at the moment [22-26]. It is therefore important to increase knowledge about the substance use-related online content that is connecting with youth and young adults.

The current study presents timely analysis of a popular Twitter handle that streams marijuana-related content. Marijuana is one of the most commonly used substances among young people in the United States. The US National Survey on Drug Use and Health (NSDUH) provides data on marijuana use across individuals ages 12 and older and the latest data indicate that past month marijuana use is highest for young adults ages 18-25 years old (18.7% in 2012 versus 19.0% in 2011) followed by 26-29 year olds (11.9% in 2012 and 12.3% in 2011) [27]. Marijuana use often begins in young adulthood with the average age being 17.9 years old in 2012.

Trends in marijuana use are important to monitor given the current shift in the marijuana policy landscape with the liberalizing of marijuana policies [28]. Currently, 19 US states and the District of Columbia now provide legal protection for the possession and supply of marijuana for medicinal purposes. A number of states and community jurisdictions have also reduced penalties for possession and use of small amounts of marijuana from criminal sanctions to fines or civil penalties. In November 2012, Colorado and Washington legalized the sale and possession of marijuana for recreational purposes. In addition, recent self-report data suggest more relaxed views toward marijuana use across both youth and adults. Specifically, population-level data indicate that most youth (60% of high school seniors) do not believe that regular marijuana use is harmful [29] and most Americans (52%) now favor legalizing the recreational use of marijuana [30].

In the US states where it is legal, medical marijuana can be used to treat various conditions including cachexia, cancer, glaucoma, human immunodeficiency virus infection/acquired immune deficiency syndrome, muscle spasms, seizures, severe nausea, severe pain, and sleep disorders [31]. Pain and muscle spasms are the most common reasons that medical marijuana is used: 89% (Arizona) and 94% (Colorado) of patients are registered for severe or chronic pain and 14% (Arizona) and 17% (Colorado) are registered for muscle spasms [32]. Nevertheless, the benefits of medical marijuana use remain uncertain with much of the evidence for marijuana’s efficacy being anecdotal [33,34]. Therefore, marijuana regulation continues to be important from a medical perspective given the known risks that are associated with its use. In 2011, marijuana contributed to over 455,000 visits to the emergency department in the United States; 13% of these patients were between the ages of 12 and 17 [35]. Additionally, there are numerous harmful short-term and long-term effects of marijuana use including short-term memory damage, impairment in attention, judgment and other cognitive functions, worsened coordination and balance, and psychotic episodes [36-39]. Persistent marijuana effects include impaired long-term memory, learning skills, and sleep, while chronic abuse can lead to addiction and increased risk for chronic cough, bronchitis, and several mental disorders including schizophrenia, anxiety, and depression [38,40].

Nevertheless, content about marijuana use is likely to have a presence on social media given its recent increased use among youth and both youths’ and adults’ more relaxed views toward marijuana use. In the present study, we assess the content of tweets and demographics of consumers who are following a popular Twitter handle (approximately 1,000,000 followers) that streams daily tweets about marijuana-related content.

## Methods

### Overview

The Twitter data in the current study is public. The Washington University Institutional Review Board reviewed our study protocol and our research was deemed exempt from human subjects review.

### Twitter Handle

**Figure 1:**
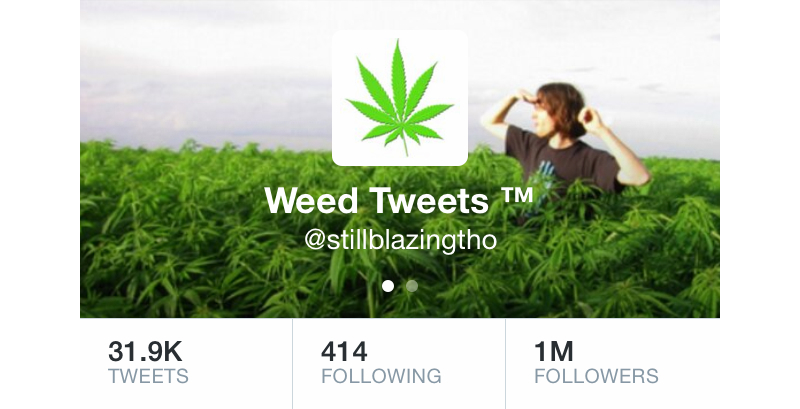
Profile summary of Weed Tweets @stillblazingtho.

We searched Twitter for popular accounts related to “marijuana” or “weed” and chose the account with the most followers: “Weed Tweets” (@stillblazingtho) with approximately 1 million followers. The next most popular marijuana-related accounts had approximately 200,000 to 300,000 followers; thus, the above account had by far the highest number of followers. The profile summary of Weed Tweets, @stillblazingtho, is shown in Figure 1.

### Tweet Engagement, Sentiment, and Content

Tweets from @stillblazingtho were collected historically for eight months (May 1, 2013-December 31, 2013). Analytics platform “SimplyMeasured” was used to access the Twitter “firehose” via Gnip (a social data firm that provides access to the Twitter “firehose” stream of every tweet ever sent) and collect all tweets sent from @stillblazingtho for the time period of interest [41]. A total of 2590 unique tweets (an average of 11 tweets per day) was sent from @stillblazingtho during the 8-month period. SimplyMeasured also provides a “Klout” score for the Twitter handle (the Klout score ranges from 1 to 100 with higher scores representing higher influence) and analysis of Twitter engagement, including the number of retweets and replies for each tweet and the number of potential impressions (total number of times a tweet from @stillblazingtho or a tweet mentioning @stillblazingtho appeared in someone’s Twitter feed during the time period).

Tweets sent from @stillblazingtho were qualitatively analyzed for sentiment and topics/themes. Tweets that were replies to another Twitter user (305/2590, 11.78% of the total tweets) were removed from the dataset because the original tweets would also need to be reviewed in order to understand the context of replies. This resulted in 2285 tweets for qualitative analysis. Tweets were coded for sentiment: positive sentiment about marijuana, negative sentiment about marijuana, neutral/unknown. Topics or themes included in tweets were additionally coded, such as whether the tweet was a joke/humorous, implied that marijuana use is not harmful or dangerous (or less harmful than other substances like alcohol), explicitly encouraged legalization, included a motivational message or quote, implied that marijuana use is good for friendship/promotes getting along, implied that you can still be successful or a good person if you use marijuana, and whether it mentioned other risky health behaviors (eg, tobacco, alcohol, other drugs, sex), the relaxing or de-stressing effects of marijuana use, frequent, regular/routine, or heavy use, blunts, marijuana edibles, or paraphernalia (eg, bongs, vaporizers), and the health benefits of marijuana or medical marijuana use. The sentiment of each tweet was coded and the topic/theme of the tweet was subsequently coded when applicable. Each tweet could be coded for more than one topic/theme if necessary.

We used crowdsourcing to code the tweets with the services of “CrowdFlower” [42]. Crowdsourcing involves using a large network of workers to complete micro-tasks. Kim et al also used crowdsourcing via CrowdFlower to analyze sentiment of tweets about US health care reform, similar to methods used for this study, and found a high level of agreement between trained coders from the research team and crowdsourced coders (82.4% for positive sentiment, 100% for negative sentiment) [43]. The tweets to be analyzed and instructions with codebook and detailed definitions (including example tweets) were provided to the CrowdFlower contributors via the online CrowdFlower platform. All tweets were coded by at least three people. Sentiment codes were a Likert scale: 1=strongly negative, 2=slightly negative, 3=neutral/unknown, 4=slightly positive, 5=strongly positive. The presence of topics/themes of interest was coded as yes/no. A set of 108 tweets (from the total 2285 tweets) coded by two trained members of the research team was considered gold standard and these were used as test questions for the CrowdFlower contributors. Only coders who scored highly on a subset of the test sample questions could begin the project. Gold standard tweets were also intermingled throughout the tweets in order to monitor coder performance throughout the project. Coders who did not perform well were dropped from the project, all prior codes from those coders were discarded, and new coders were assigned in their place.

Because tweets were coded by multiple coders, the numeric values for sentiment coding were first averaged and then collapsed into negative (values 1 to 2.4), neutral/unknown (values 2.5 to 3.4), and positive (3.5 to 5.0). For the yes/no items, the response from the most “trusted” coder (based on coding accuracy compared to gold standard questions) was chosen; when “trust” scores among the coders were close, the most popular response was chosen. Based on our own coding of 108 test questions compared to final codes from CrowdFlower contributors, overall level of agreement was high. Percent agreement was 91% for sentiment, and ranged from 76% to 100% for topic codes (76% was for the joke/humorous code, which would be expected to have lower agreement due to the subjective nature of the code).

Hashtags (symbol #) are used before a relevant keyword or phrase in a tweet to categorize the tweet so that people can find them more easily in their Twitter search. We also extracted tweets that included hashtags and two members of the research team coded the hashtags as being related to marijuana or not related to marijuana.

### Demographics of Followers

We used “Demographics Pro for Twitter” [44], described in detail below, to report on the predicted demographic characteristics of followers of @stillblazingtho and the characteristics of the average Twitter user. Inferred characteristics of followers included geographic location, gender, marital status, age, race, income, occupation, other likes and interests, and other Twitter handles followed. We also report on the followers’ level of Twitter activity (eg, number of tweets/day, number of handles followed, number of their own followers), which is not inferred or predicted but rather taken from explicit Twitter data or metadata.

Inferred demographics data on current followers of @stillblazingtho on December 9, 2013 at 2:30pm EST were obtained from Demographics Pro [44], which provides analysis of followers of Twitter accounts for a fee. Demographics Pro estimates demographic characteristics based on Twitter behavior/usage, relying on multiple data signals from networks (signals imparted by the nature and strength of ties between individuals on Twitter), consumption (consumption of information on Twitter revealed by accounts followed and real-world consumption revealed by Twitter usage), and language (words and phrased used in tweets and bios). A random sample of 50,000 followers of @stillblazingtho was analyzed, regardless of whether they posted or commented to @stillblazingtho. The data signals were filtered and amplified using large proprietary knowledge bases of established correlations between data points and demographic characteristics. The multiple amplified signals were combined using a series of algorithms to estimate or infer the likely demographic characteristics. Demographics Pro has used their methodology to profile some 300 million Twitter users to date. The methodologies used in the prediction of demographic characteristics of Twitter followers include big data, natural language processing, entity identification, image analyses, and network theory. Demographics Pro requires confidence of 95% or above to make an estimate of a single demographic characteristic [44]. For example, if 10,000 predictions are made, 9500 would need to be correct in order to accept the methodology used to make the prediction. The success of the Demographics Pro analytic predictions relies on the relatively low covariance of multiple amplified signals. Iterative evaluation testing the methodologies on training sets of established samples of Twitter users with verified demographics allows the calibration of balance between depth of coverage (the number of demographic predictions made) and required accuracy. The size of these established samples of Twitter users with verified demographics varies from 10,000 to 200,000 people depending on the specific demographic characteristic to be inferred. For comparison purposes, Demographics Pro also reports the distributions of the median average and inter-quartile range [IQR] for follower demographic characteristics across a sample of approximately 250,000 Twitter accounts from 10 million Twitter accounts analyzed by Demographics Pro. Inter-quartile ranges are not presented for age or income because the median averages for these categorical variables are weighted so that the sum of the weighted medians over all categories totals 100%.

Characteristics of @stillblazingtho followers were descriptively compared to the median average of the characteristics distributions for Twitter users. Finally, we also report on the popularity of the @stillblazingtho Twitter account within demographic groups based on rankings by Demographics Pro. To examine the popularity of the Twitter handle of interest within demographic groups, Demographics Pro ranks a subset (approximately 250,000 handles with 1000 or more followers) of the 10,000,000 Twitter handles they have analyzed by number of followers within specific demographic groups.

## Results

### Tweet Engagement, Sentiment, and Content

A total of 2590 tweets (2285 regular tweets and 305 replies) were sent from @stillblazingtho from May 1, 2013 to December 31, 2013 (average of 11 tweets per day). The Klout score for @stillblazingtho was 77.8. Regarding engagement, there were a total of 1,964,908 retweets of @stillblazingtho tweets and 135,797 replies to @stillblazingtho during the 8-month time period. Total potential impressions, or total number of times a tweet from @stillblazingtho or a tweet mentioning @stillblazingtho appeared in someone’s Twitter feed, was 2,898,866,761 during the 8-month period.

Qualitative analysis was performed on the 2285 regular tweets sent from @stillblazingtho (305 replies representing 11.78% of total tweets were excluded). Of these tweets that excluded replies, 1875 (82.06%) were positive about marijuana, 403 (17.64%) were either neutral in sentiment or were not specifically about marijuana, and 7 (0.31%) appeared negative about marijuana. Percentages for sentiment of tweets included in the qualitative analysis (excluding replies) and also among total tweets (including replies) are presented in Table 1.

**Figure 2:**
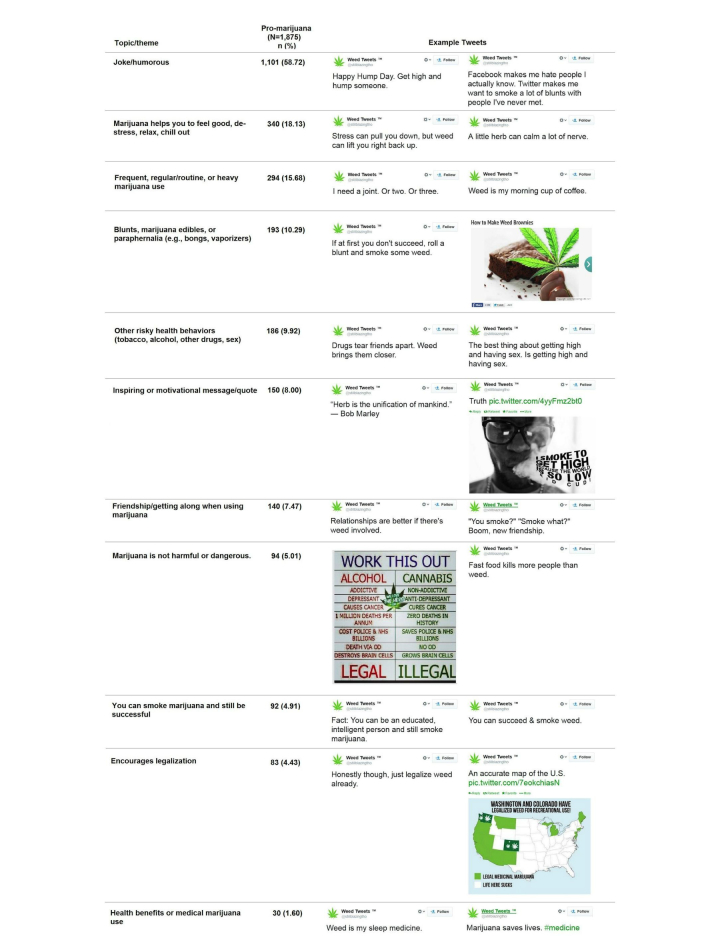
Topics and themes present in positive marijuana Tweets.

The distribution of specific topics for the positive marijuana tweets along with example tweets are presented in Figure 2. Most of the positive marijuana tweets were viewed as jokes or humorous (1101/1875, 58.72%) followed by tweets that implied that marijuana helps you to feel good, relax, or chill (340/1875, 18.13%); 15.68% (294/1875) of the tweets mentioned routine, frequent, or heavy use, and 193 (10.29%) mentioned blunts, marijuana edibles, or paraphernalia (eg, bongs, vaporizers). Approximately 186 (9.92%) of the 1875 positive marijuana tweets mentioned other risky health behaviors (eg, tobacco, alcohol, other drugs, sex). Additional results are shown in Figure 2.

Of the 403 neutral tweets, 70 (17.4%) were inspirational or motivational quotes/messages and 58 (14.4%) were jokes/humorous; for example, “If you are always worried about what others think of you, you will never be happy” or “Sitting there wondering why someone hasn’t texted you back, and realizing you never finished sending the message”. Examples of the seven negative tweets include, “If you smoke weed to be cool, you’re a fucking loser” or “I know too many people who have died from drug overdoses. When the fuck are people going to learn #RIP”.

Of the total 2590 tweets sent from @stillblazingtho, 135 (5.21%) contained the use of a hashtag. Only 26 (19.26%) of these hashtags were marijuana specific (eg, #weed, #staystoned, and #stayhigh), while tweets including general hashtags that were non-marijuana related were 109 (80.74%) (eg, #ThingsIWillTeachMyChild, #firstdayofsummer, and #TheSecretToLifeIs).

### Demographics of @stillblazingtho Followers

Characteristics of @stillblazingtho followers, other than Twitter activity (eg, tweets per day, number of followers, number of accounts followed), were inferred by Demographics Pro. Of the 959,143 followers of @stillblazingtho, 759,407 (79.17%) were in the United States, 60,211 (6.28%) in the United Kingdom, 41,716 (4.35%) in Canada, and <1% in each of South Africa (n=5460; 0.88%), Netherlands (n=7785; 0.81%), and Mexico (n=6885; 0.72%). Within the United States, @stillblazingtho was in the top 20% of all Twitter accounts. The Twitter followers were active: 34.01% (326,242/959,143) had >5 tweets/day and 36.71% (352,148/959,143) had 1-5 tweets/day. Approximately 82.19% (788,310/959,143) followed a total of 101-1000 of Twitter accounts, and 68.34% (655,489/959,143) of users had a high number of their own followers (101-1000). A total of 54.03% (518,184/959,143) of @stillblazingtho followers were female, which is similar to the Twitter median average (52.6%, IQR 40.7-67.6%). Approximately 81.14% (778,240/959,143) of the followers were single, compared to the Twitter median average of only 38.1% (IQR 9.5-75.1%).

**Figure 3:**
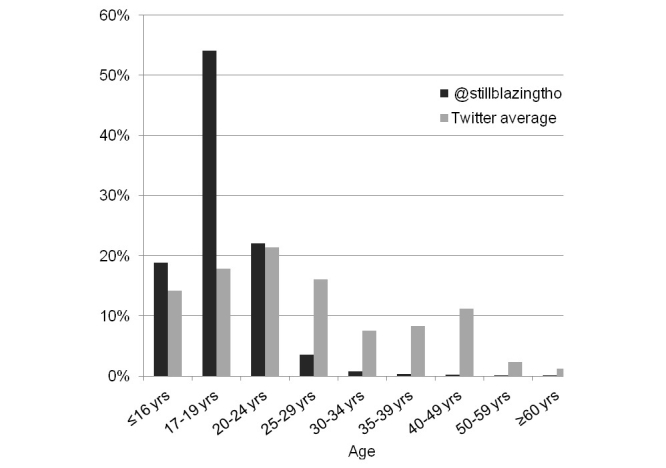
Age distribution of @stillblazingtho followers and Twitter median average.

Followers of @stillblazingtho were younger than the Twitter median average age distribution (Figure 3). Most followers of @stillblazingtho were 17-19 years old (518,430/959,143; 54.05%); 18.84% (180,673/959,143) were 16 years old or younger, 22.0% (210,799/959,143) were 20-24 years old, and only 5.11% (49,047/959,143) were 25 years old or older. The Twitter median average age distribution was: 14.2% were 16 years old or younger, 17.8% were 17 to 19 years old, 21.4% were 20-24 years old, 16.0% were 25-29 years old, 15.8% were 30-39 years old, 11.2% were 40-49 years old, and 3.5% ≥50 years old. Among people aged 17 to 19 years, @stillblazingtho was in the top 10% of all Twitter accounts followed.

**Figure 4:**
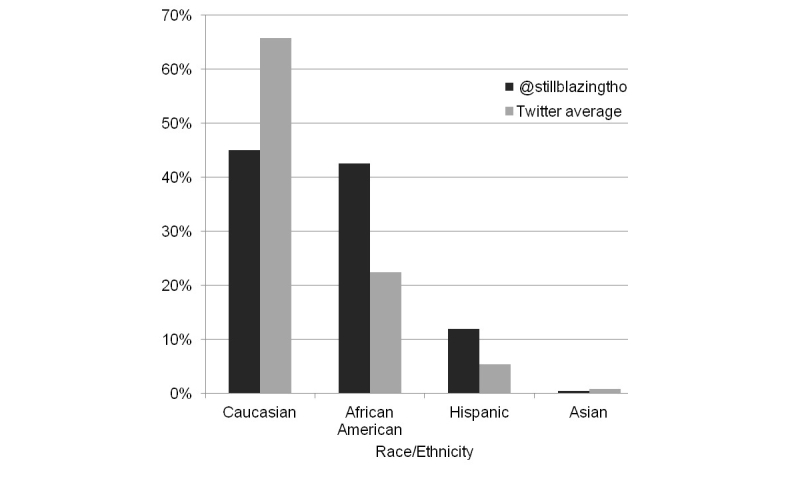
Race/ethnicity distribution of @stillblazingtho followers and Twitter median average.

More followers of @stillblazingtho in the United States were African American (323,107/759,407; 42.55%) or Hispanic (90,732/759,407; 11.95%) than the Twitter median average (African American 22.4%, IQR 5.1-62.5%; Hispanic 5.4%, IQR 3.0-10.8%) (Figure 4). Among Hispanics, @stillblazingtho was in the top 30% of all Twitter accounts followed. Personal income among all followers of @stillblazingtho was somewhat lower than the Twitter median average, with 93.53% (897,041/959,143) under US$30,000 per year (Twitter median average 76.9% under $30,000 per year).

More @stillblazingtho followers were students (267,855/959,143; 27.93%) and musicians (205,967/959,143; 21.47%) than the Twitter median average (9.1% students, IQR 4.9-15.0%; 8.2% musicians, IQR 3.3-17.7%). Among students and musicians, @stillblazingtho was in the top 10% and top 20%, respectively, of all Twitter accounts. Music (290,228/959,143; 30.26%) and basketball (274,514/959,143; 28.62%) were the most common interests of @stillblazingtho followers, compared to Twitter median averages of 14.0% music (IQR 9.0-22.9%) and 10.1% basketball (IQR 4.6-19.7%). Many followers of @stillblazingtho also followed rappers and recording artists such as Wiz Khalifa (453,477/959,143; 47.28%), Drake (327,645/959,143; 34.16%), Lil Wayne (323,616/959,143; 33.74%), Mac Miller (277,592/959,143; 28.94%), Nicki Minaj (256,961/959,143; 26.79%), Rihanna (248,927/959,143; 25.95%), and Eminem (234,884/959,143; 24.49%). Twitter median averages for the above recording artists ranged from 7.0% (for Mac Miller, IQR 4.9-8.8%) to 12.6% (for Rihanna, IQR 5.6-24.5%).

**Table 1:** Characteristics of “Weed Tweets @stillblazingtho” tweets, 5/1/2013-12/31/2013.

Sentiment of tweets^a^	Total tweets	Replies	Tweets excluding replies
	n=2590	n=305	n=2285
n (%)	n (%)		n (%)
Positive	1875 (72.39)	-	1875 (82.06)
Neutral	403 (15.56)	-	403 (17.64)
Negative	7 (0.27)	-	7 (0.31)

^a^Sentiment of tweets was determined only for regular tweets. Direct replies were excluded because the context of the conversation was difficult to determine without additional information.

## Discussion

### Principal Findings

The @stillblazingtho is a popular Twitter handle with approximately 1 million followers. This Twitter handle sends an average of 11 tweets per day, the vast majority of which promote marijuana use. Most tweets generated from @stillblazingtho contain humorous content about marijuana use followed by tweets that suggested that marijuana helps you to feel good, relax, or chill. This Twitter handle encourages favorable attitudes toward marijuana by distributing a high number of tweets normalizing the routine use of marijuana and promoting its relaxation effects. It additionally engages followers about pro-marijuana culture by tweeting about such content as marijuana edibles (eg, recipes for brownies) and paraphernalia commonly used to smoke marijuana, like bongs and vaporizers. Tweets that minimize the harmful effects of marijuana use and associate its use with health benefits and/or stronger peer relationships are also distributed by @stillblazingtho. In addition, tweets that encourage the legalization of marijuana are sent by this Twitter handle, but this is done to a lesser degree. While tweets from @stillblazingtho comprised a number of themes and topics, most tweets were alike in their overarching positive sentiment toward marijuana use.

The majority of the followers of @stillblazingtho who are being exposed to this pro-marijuana content are predicted to be under 20 years of age (approximately 73%) and 19% are under 17 years old. The average age at which marijuana use begins in the United States is currently at 17.9 years old [27]; therefore, our results call attention to the majority of Twitter followers of @stillblazingtho who are either approaching or are very near the average age at which marijuana use is first initiated. Moreover, young people are especially responsive to social media influences and often establish substance use patterns during this phase of development [19-21]. Thus, it is of concern that so many youth and young adults are following a Twitter handle that depicts marijuana use as a popular and normal social activity. In addition, past research has found that young Twitter users can become exposed to tweets promoting alcohol use via interactive features such as hashtags on other unrelated sites [45-46]. The extent of hashtags in tweets from @stillblazingtho was relatively low. Nevertheless, the inclusion of general hashtags (non-marijuana related) in any of the tweets sent by this Twitter handle have the potential to reach a much wider audience of youth and young adults beyond the followers that we analyzed in the current study.

Another primary finding of our study is that African American and Hispanic Twitter users disproportionately follow @stillblazingtho versus Caucasians. This finding signals a disparity in exposure to social media promoting marijuana use in that the pro-marijuana tweets delivered by this handle are disproportionately consumed by minority Twitter users. Our findings match concerning differences in marijuana use by race/ethnicity reported in previous studies [47-49]. The frequency of marijuana abuse and dependence among African American adults is about twice the rate of Caucasians and Hispanics [50]. With regard to Hispanics, marijuana abuse and dependence rates are closer to the rates of Caucasians, but the latest reports show that Hispanic youth now have the highest rates of marijuana use versus Caucasians and African Americans [51]. Accordingly, our findings underscore the critical need to improve understanding on how African Americans and Hispanics engage with social media outlets like Twitter in ways that may exacerbate their marijuana use.

The @stillblazingtho followers receive pro-marijuana use content from this Twitter handle and could be receiving similar marijuana-related content from other handles. For instance, many of the @stillblazingtho followers are alike in that they follow the same celebrity Twitter handles. One or more of these celebrities could also be tweeting favorably about recreational marijuana use. To illustrate this point, we provide a sample tweet from Wiz Khalifa who is a recording artist followed by many of @stillblazingtho followers (47.3%). On February 8, 2014, Wiz Khalifa tweeted, “Those who don’t understand the beauty of weed, purchasing weed, rolling and sharing of weed are outsiders and have no business in our world.” This tweet demonstrates the likelihood for pro-marijuana content to be distributed by multiple Twitter handles to a cluster of followers. A study of all the pro-marijuana content that is being consumed by the followers of @stillblazingtho is beyond the scope of this study; nevertheless, it is important for public health professionals to consider all of the tweets and Twitter handles that promote harmful norms toward substance use and are connecting with young people. Prevention efforts can use this information to connect with Twitter users in a strategic and meaningful way. One such strategy would be for public health professionals to consider partnering with a popular celebrity who is willing to tweet health promoting messages about the harms associated with marijuana use. Likewise, many of the followers of @stillblazingtho are students and/or musicians, and have interests in music and basketball. Perhaps, these data could be used to distinguish persons who are at increased risk for marijuana use and/or to identify appropriate settings where marijuana use prevention messages could be delivered (eg, music concerts).

### Limitations

Some limitations should be considered when interpreting the results. First, demographics of followers are not actual reported demographics but rather inferred based on Twitter behavior/usage. However, Demographics Pro uses sophisticated methodology (reported in the Methods section) to make such inferences and requires confidence of 95% or above to make an estimate of a single demographic characteristic [44]. Second, we report on only one of many marijuana-related Twitter handles. Demographics of other specific marijuana-related handles could differ from the one we chose to analyze. Nevertheless, we reported on a very popular marijuana-related Twitter handle, whose followers greatly outnumbered those of other handles. Our study did not examine Twitter marijuana discourse in a general way, where both favorable and unfavorable tweets are considered in analysis. Such a study would entail a data collection and analysis of countless tweets that contain any and all marijuana-related terms, and is beyond the scope of our study. We nevertheless encourage future studies to work toward understanding marijuana-related communication on Twitter utilizing a more general approach where both favorable and unfavorable content is considered. Finally, we have no way of inferring whether followers of @stillblazingtho are themselves marijuana users or are non-marijuana users. Non-marijuana users might be different from marijuana users in their reasons for following @stillblazingtho; it is, therefore, challenging to make broad-stroke conclusions about why the followers of @stillblazingtho have opted to receive tweets from this handle.

### Conclusions

Despite these limitations, our results stress the need for continued research and surveillance on the pro-marijuana content that is currently being delivered via Twitter. We found that youth and young adults as well as minority Twitter users are disproportionately more likely to follow @stillblazingtho, which is a popular Twitter handle that distributes a high number of tweets encouraging favorable attitudes toward marijuana use. Our findings provide a snapshot of the pro-marijuana content that is reaching young people. Twitter use has expanded exponentially, especially among youth and young adults; therefore, an improved understanding of the discourse on Twitter that encourages marijuana use can be helpful for tailoring and targeting online and offline prevention messages.

## Abbreviations

IQR: inter-quartile range
MPM: Media Practice Model
